# Vitamin D supplementation for prevention of acute respiratory infections in older adults: A systematic review and meta-analysis

**DOI:** 10.1371/journal.pone.0303495

**Published:** 2024-05-24

**Authors:** Hao Jia, Feng Sheng, Yulan Yan, Xiaozhi Liu, Baoqi Zeng

**Affiliations:** 1 Drug Clinical Trial Institution, Tianjin Fifth Central Hospital (Peking University Binhai Hospital), Tianjin, China; 2 Department of Education and Science, Tianjin Fifth Central Hospital (Peking University Binhai Hospital), Tianjin, China; 3 Central Laboratory, Tianjin Fifth Central Hospital (Peking University Binhai Hospital), Tianjin, China; 4 Tianjin Key Laboratory of Epigenetic for Organ Development of Preterm Infants, Tianjin Fifth Central Hospital, Tianjin, China; 5 High Altitude Characteristic Medical Research Institute, Huangnan Tibetan Autonomous Prefecture People’s Hospital, Huangnan Prefecture, Qinghai, China; 6 Emergency Department of Tianjin Fifth Central Hospital, Tianjin, China; 7 Department of Epidemiology and Biostatistics, School of Public Health, Peking University Health Science Centre, Beijing, China; The University of Sydney, AUSTRALIA

## Abstract

**Background:**

Acute respiratory infections (ARIs) have a substantial impact on morbidity, healthcare utilization, and functional decline among older adults. Therefore, we systematically reviewed evidence from randomized controlled trials (RCTs) to evaluate the efficacy and safety of vitamin D supplementation in preventing ARIs in older adults.

**Methods:**

PubMed, Embase, the Cochrane Library, and ClinicalTrials.gov were searched until 1 February 2024. RCTs evaluating the use of vitamin D supplements to protect older adults from ARIs were included. Two reviewers independently screened papers, extracted the data and assessed the risk of bias. Data were summarised as relative risks (RRs) or odds ratios (ORs) with corresponding 95% confidence intervals (CIs). Random effects meta-analyses were used to synthesise the results. GRADE was used to evaluate the quality of evidence. All the analysis were performed with Stata version 17.

**Results:**

Twelve trials (41552 participants) were included in the meta-analysis. It showed that vitamin D supplementation probably does not reduce the incidence of ARIs (RR, 0.99; 95% CI, 0.97–1.02, I^2^ = 0%; moderate certainty). No significant effect of vitamin D supplementation on the risk of ARI was observed for any of the subgroups defined by baseline 25(OH)D concentration, control treatments, dose frequency, study duration, and participants’ condition. However, there was a possibility, although not statistically significant, that vitamin D may reduce the risk of ARI in patients with a baseline 25(OH)D concentration <50 nmol/L (OR, 0.90; 95% CI, 0.79–1.04, I^2^ = 14.7%). Additionally, vitamin D supplements might result in little to no difference in death due to any cause, any adverse event, hypercalcinemia, and kidney stones.

**Conclusions:**

Vitamin D supplementation among older adults probably results in little to no difference in the incidence of ARIs. However, further evidence is needed, particularly for individuals with vitamin D deficiency and populations residing in low and middle income countries.

**Trial registration:**

*This study was registered on PROSPERO (CRD42023451265)*.

## 1 Introduction

Acute respiratory infections (ARIs) are common, and many individuals experience them at least once a year. These infections affect various parts of the respiratory system, including the nose, throat, sinuses, and lungs [1,2]. ARIs are classified into upper respiratory tract infections (URIs) and lower respiratory tract infections (LRIs). URIs primarily affect the upper respiratory system, including the nasal passages, sinuses, throat, and larynx, while LRIs involve the lower respiratory system, including the trachea, bronchi, and lungs. LRI is a leading cause of morbidity and mortality around the world [1].

Vitamin D, or calciferol, is a fat-soluble vitamin that primarily supports bone health. The active form of vitamin D, called 1,25-dihydroxy vitamin D (1,25(OH)2D), may enhance innate immunity and exert anti-inflammatory effects [3]. Interest in the potential of vitamin D supplementation to reduce the risk of ARIs has surged following the onset of the COVID-19 pandemic [4]. Pre-clinical studies have indicated that vitamin D supplementation plays a crucial role in supporting immune function [5,6]. Furthermore, observational studies have consistently shown an inverse association between serum 25(OH)D levels and the risk or severity of ARIs [7,8]. However, randomized controlled trials (RCTs) of vitamin D supplementation for the prevention of ARI have produced heterogeneous results, with some showing protection, and others reporting null findings [9]. Also, findings from meta-analyses of RCTs of vitamin D supplementation have produced inconsistent results [9–12]. It is important to note that the previous meta-analyses [9–12] included participants across different age groups, including pediatric, older adults, and mostly healthy adults. Although previous review has conducted a basic subgroup analysis based on participants’ age [10], there is a need for a comprehensive analysis of the efficacy and safety outcomes of vitamin D supplementation, particularly for vulnerable populations, such as children and older adults.

While some meta-analyses of the association between vitamin D supplementation and the risk of ARIs in the pediatric populations have been published [13,14], the efficacy of vitamin D supplementation in preventing ARIs in older adults remains uncertain and has not been thoroughly reviewed to the best of our knowledge. The number of older adults and adults with age-related chronic diseases is projected to more than double between 2019 and 2050 [15]. ARIs are prevalent among older adults and have a substantial impact on morbidity, healthcare utilization, and functional decline [1]. Therefore, we systematically reviewed evidence from RCTs to evaluate the efficacy and safety of vitamin D supplementation in preventing ARIs in older adults.

## 2 Materials and methods

### 2.1 Search strategy and selection criteria

We conducted a search for published literature on databases such as PubMed, Embase, and the Cochrane Library, as well as on the trial registry ClinicalTrials.gov up to February 1, 2024. The search strategies were developed and piloted by the review team for bibliographic databases and clinical trial registry using medical subject headings or Emtree and key words for “vitamin D”, “acute respiratory infection”, and “randomized controlled trials”. The detailed search strategy was shown in the S1 File. We conducted this meta-analysis according to the Preferred Reporting Items for Systematic reviews and Meta-Analyses (PRISMA) guidelines [16], and the protocol was registered on PROSPERO (CRD42023451265).

RCTs (including cluster RCTs) evaluating the effects of supplementary vitamin D3, vitamin D2, or 25(OH)D, regardless of dosage or duration, to prevent ARI in adults 50 years of age or older were included. If the mean age of all participants was 60 years or older, the study was also included. The control group could be a placebo or low-dose vitamin D. To minimize misclassification bias, only studies with ARI as a prespecified efficacy outcome were included. Our search was restricted to papers published in English. Conference abstracts, ongoing trials, and studies lacking sufficient data for analysis were excluded. Two reviewers (HJ and FS) independently screened the titles and abstracts of all identified records using Endnote X9. We retrieved full-length records of those deemed eligible and screened these again to confirm inclusion. Disagreements were resolved by discussion, or with the help of a third reviewer (BZ) when consensus could not be reached.

### 2.2 Data extraction

Two reviewers (HJ and YY) independently extracted data on the study characteristics, patient characteristics, interventions, comparisons, and outcomes from each study using a standardized piloted form in Excel. The primary outcome was the proportion of participants who had one or more ARIs, with the definition of ARI encompassing events classified as upper respiratory infection, lower respiratory infection, and ARI in an unclassified location. Secondary outcomes were the proportions of participants experiencing one or more of the following outcomes: upper respiratory infection; lower respiratory infection; hospital admission due to an ARI; death due to any cause; any adverse event; and potential adverse reactions to vitamin D (hypercalcinemia and kidney stones).

### 2.3 Risk of bias and certainty of evidence

The risk of bias of RCTs was assessed by the Cochrane Collaboration’s tool (RoB 2) [17]. Assessment was made across five domains of bias (randomization process, deviations from intended interventions, missing outcome data, measurement of the outcome, and selection of the reported result). Risk of bias was assessed as either low (proper methods taken to reduce bias), high (inadequate methods creating bias), or some concerns for each of the five domains of bias. The quality of evidence for each outcome was evaluated with the Grading of Recommendations Assessment, Development and Evaluation (GRADE) framework [18].

### 2.4 Data synthesis and analysis

We conducted DerSimonian and Laird random-effects meta-analysis when data were available for at least two studies [19]. Outcomes were reported as relative risks (RRs) or odds ratios (ORs) with corresponding 95% confidence intervals (CIs). Statistical heterogeneity between the studies was assessed with the Cochran’s χ^2^ test and the I^2^ statistics. I^2^ values of 25%, 50%, and 75% have been suggested to be indicators of low, moderate, and high heterogeneity, respectively [20]. We also conducted subgroup analysis based on baseline 25(OH)D concentration (<50 nmol/L vs ≥ 50 nmol/L), control treatments (placebo versus lower-dose vitamin D supplementation), dose frequency (daily vs bolus vs combination), study duration (≤ 1 year vs > 1 year), and participants’ condition (healthy vs comorbidity). Publication bias was assessed using a funnel plot for the primary outcome. Finally, we performed sensitivity analyses for primary outcome by excluding the studies considered at high risk of bias and excluding each individual study to evaluate its influence on the overall findings. All the analysis were performed with Stata version 17.

## 3 Results

### 3.1 Characteristics of included studies

**Fig 1:**
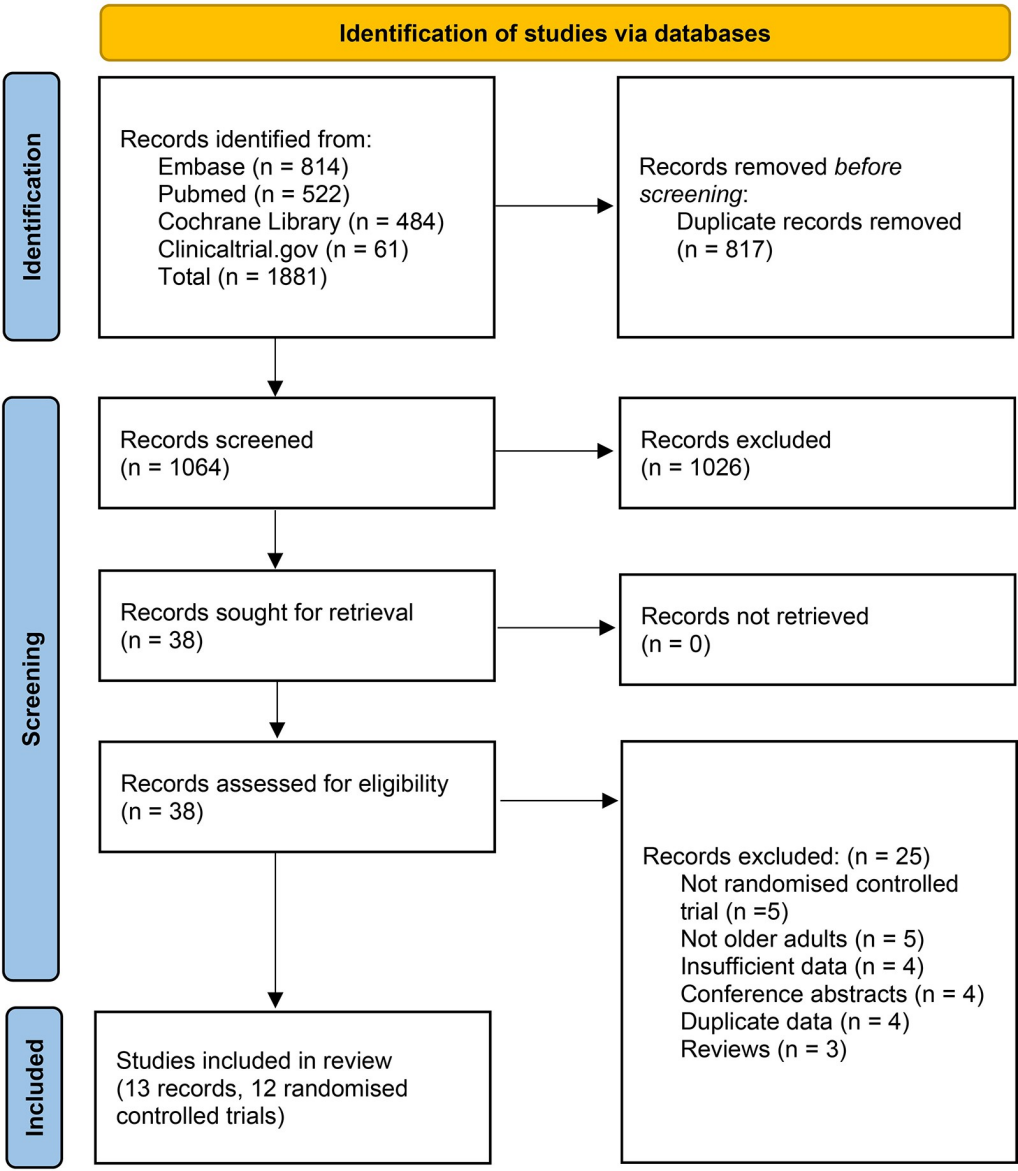
Study selection.

This systematic literature search initially identified 1881 records, after excluding duplicates and irrelevant papers, 38 papers were evaluated in full text for eligibility (Fig 1). Finally, 12 RCTs (43118 participants) were included in the present meta-analysis [21–33]. Ten studies compared the effects of one vitamin D regimen with placebo only [21,23,24,26–33], and two studies compared the effects of higher-dose vitamin D with lower-dose vitamin D regimens [22,25]. Vitamin D was given as bolus doses once per month to once every 2 months in 6 studies [21,23,27–30]; as daily doses in 4 studies [24,26,32,33]; and as a combination of bolus and daily doses in 2 studies [22,25]. Trial durations ranged from 3 months to 5 years. All the trials included were conducted exclusively in high-income countries. Participants’ mean age and mean 25(OH)D concentrations at baseline ranged from 61.2 to 80.7 years and from 41.7 to 77.4 nmol/L, respectively. Characteristics of individual studies are summarised in Table 1.

**Table 1:** Summary of included randomised controlled trials.

Study, year	Participants, female (%)	Mean age (SD; range)	Baseline 25(OH)D, nmol/L (SD)	Intervention group	Control group	Study duration
Camargo, 2020 [23]	5110, 41.5%	66.4 (8.3; 50.0–84.0)	63.4 (23.6)	VD3 (one 5000 μg bolus loading dose, then 2500 μg bolus monthly)	Placebo	3 years
Tran, 2014 [28]	644, 46.7%	71.7 (6.9; 60.3–85.2)	41.7 (13.5)	VD3 (750 μg or 1500 μg bolus monthly)	Placebo	1 year
Martineau, 2015 (ViDiFlu) [25]	240, 65.8%	67.1 (13.0; NR)	42.9 (23.0)	VD3 (older adults, 2400 μg bolus once every 2 months plus 10 μg daily; carers, 3000 μg once every 2 months)	Older adults, placebo plus 10 μg VD3 daily; carers, placebo	1 year
Aloia, 2019 [24]	260, 100%	69.0 (5.3; 65.4–72.5)	54.4 (16.7)	VD3 (50 μg daily)	Placebo	3 months
Bischoff-Ferrari, 2020 [26]	2157, 61.7%	74.9 (4.4; 70.0–95.0)	55.9 (21.0)	VD3 (50 μg daily)	Placebo	3 years
D-Health Trial [21,31]	16000, 45.8%	69.3 (5.5; 60.0–86.0)	NA	VD3 (1500 μg bolus monthly)	Placebo	5 years
Ginde, 2017 [22]	107, 57.9%	80.7 (9.9; 60.0–95.0)	57.3 (22.7)	VD3 (2500 μg bolus monthly plus ≤ 25 μg per day equivalent)	Placebo plus 10–25 μg VD3 per day equivalent	1 year
Rake, 2020 [27]	1615, 47.1%	72.2 (4.9; 65.0–84.0)	50.2 (27.1)	VD3 (2500 μg bolus monthly)	Placebo	2 years
Lehouck, 2012 [30]	182, 19.8%	67.9 (8.3; 48.0–86.0)	49.8 (29.2)	VD3 (2500 μg bolus monthly)	Placebo	1 year
Rees, 2013 [33]	759, 42.3%	61.2 (6.6; 47.1–77.9)	62.5 (21.3)	VD3 (25 μg daily)	Placebo	1 year
Martineau, 2015 (ViDiCO) [29]	240, 40.0%	64.7 (8.5; 40.0–85.0)	46.1 (25.7)	VD3 (3000 μg bolus once every 2 months)	Placebo	1 year
Camargo, 2023 [32]	15804, 51%	68.0 (7.0; 50.0–NR)	77.4 (24.9)	VD3 (50 μg daily)	Placebo	1 year

SD, standard deviation; NA, not reported; VD3, vitamin D3.

### 3.2 Risk of bias and certainty of evidence

Seven of the 12 trials were classified as low risk of bias (41552 patients), three trials had some concerns (1144 patients), and two trials (422 patients) was classified as high risk of bias owing to deviation from intended intervention and missing outcome data. Risk of bias assessments in individual studies, including reasons, are listed in the characteristics of included studies in Table 2. The quality of evidence for each outcome was rated following the GRADE framework (Table 3).

**Table 2:** Risk of bias for included RCTs.

Study	Randomization process	Deviations from intended interventions	Missing outcome data	Measurement of the outcome	Selection of the reported result	Overall bias
Camargo, 2020 [23]	Low	Low	Low	Low	Low	Low
Tran, 2014 [28]	Some concerns	Low	Low	Low	Low	Some concerns
Martineau, 2015 (ViDiFlu) [25]	Low	High	High	Low	Low	High
Aloia, 2019 [24]	Low	Low	Some concerns	Low	Low	Some concerns
Bischoff-Ferrari, 2020 [26]	Low	Low	Low	Low	Low	Low
D-Health Trial	Low	Low	Low	Low	Low	Low
Ginde, 2017 [22]	Low	Low	Low	Low	Low	Low
Rake, 2020 [27]	Low	Low	Low	Low	Low	Low
Lehouck, 2012 [30]	Low	Some concerns	High	Low	Low	High
Rees, 2013 [33]	Low	Low	Low	Low	Low	Low
Martineau, 2015 (ViDiCO) [29]	Low	Some concerns	Low	Low	Low	Some concerns
Camargo, 2023 [32]	Low	Low	Low	Low	Low	Low

**Table 3:** GRADE assessment.

Outcomes	Quality assessment								No of trials	Effects	GRADE
	Study limitations	Inconsistency	Indirectness	Imprecision	Reporting bias	Large effect	Dose response	Other Factors			
ARI	***Serious*** ^**a**^	*Not serious*	*Not serious*	*Not serious*	*Not serious*	*None*	*None*	*None*	12	RR, 0.99; 95% CI, 0.97–1.02, I^2^ = 0	**Moderate**
URI	***Serious*** ^**a**^	*Not serious*	*Not serious*	*Not serious*	*Not serious*	*None*	*None*	*None*	8	RR, 1.00; 95% CI, 0.97–1.03, I^2^ = 2.0%	**Moderate**
LRI	***Serious*** ^**a**^	*Not serious*	*Not serious*	*Not serious*	*Not serious*	*None*	*None*	*None*	4	RR, 0.99; 95% CI, 0.91–1.07, I^2^ = 0	**Moderate**
Hospital admission due to an ARI	***Serious*** ^**a**^	*Not serious*	*Not serious*	*Not serious*	*Not serious*	*None*	*None*	*None*	4	RR, 0.98; 95% CI, 0.87–1.09, I^2^ = 0	**Moderate**
Death due to any cause	***Serious*** ^**a**^	*Not serious*	*Not serious*	***Serious*** ^**d**^	*Not serious*	*None*	*None*	*None*	6	RR, 1.11; 95% CI, 0.75–1.64, I^2^ = 1.3%	**Low**
Any adverse event	***Serious*** ^**a**^	***Serious*** ^**b**^	*Not serious*	*Not serious*	*Not serious*	*None*	*None*	*None*	6	RR, 0.94; 95% CI, 0.87–1.03, I^2^ = 75.7%	**Low**
Hypercalcinemia	*Not serious*	*Not serious*	*Not serious*	***Serious*** ^**c**^	*Not serious*	*None*	*None*	*None*	2	RR, 1.14; 95% CI, 0.71–1.81, I^2^ = 22.8%	**Moderate**
Hidney stones	*Not serious*	*Not serious*	*Not serious*	***Serious*** ^**c**^	*Not serious*	*None*	*None*	*None*	2	RR, 0.99; 95% CI, 0.78–1.27, I^2^ = 0%	**Moderate**

ARI, acute respiratory infection; RR, relative risk; URI, upper respiratory tract infection; LRI, lower respiratory tract infection; CI, confidence interval; GRADE, Grading of Recommendations, Assessment, Development, and Evaluation criteria.

^a^ Include trials with high-risk bias or some concerns.

^b^ High heterogeneity.

^c^ Only two trials were included.

^d^ The 95% CI was imprecise.

### 3.3 Meta-analysis of ARIs

**Fig 2:**
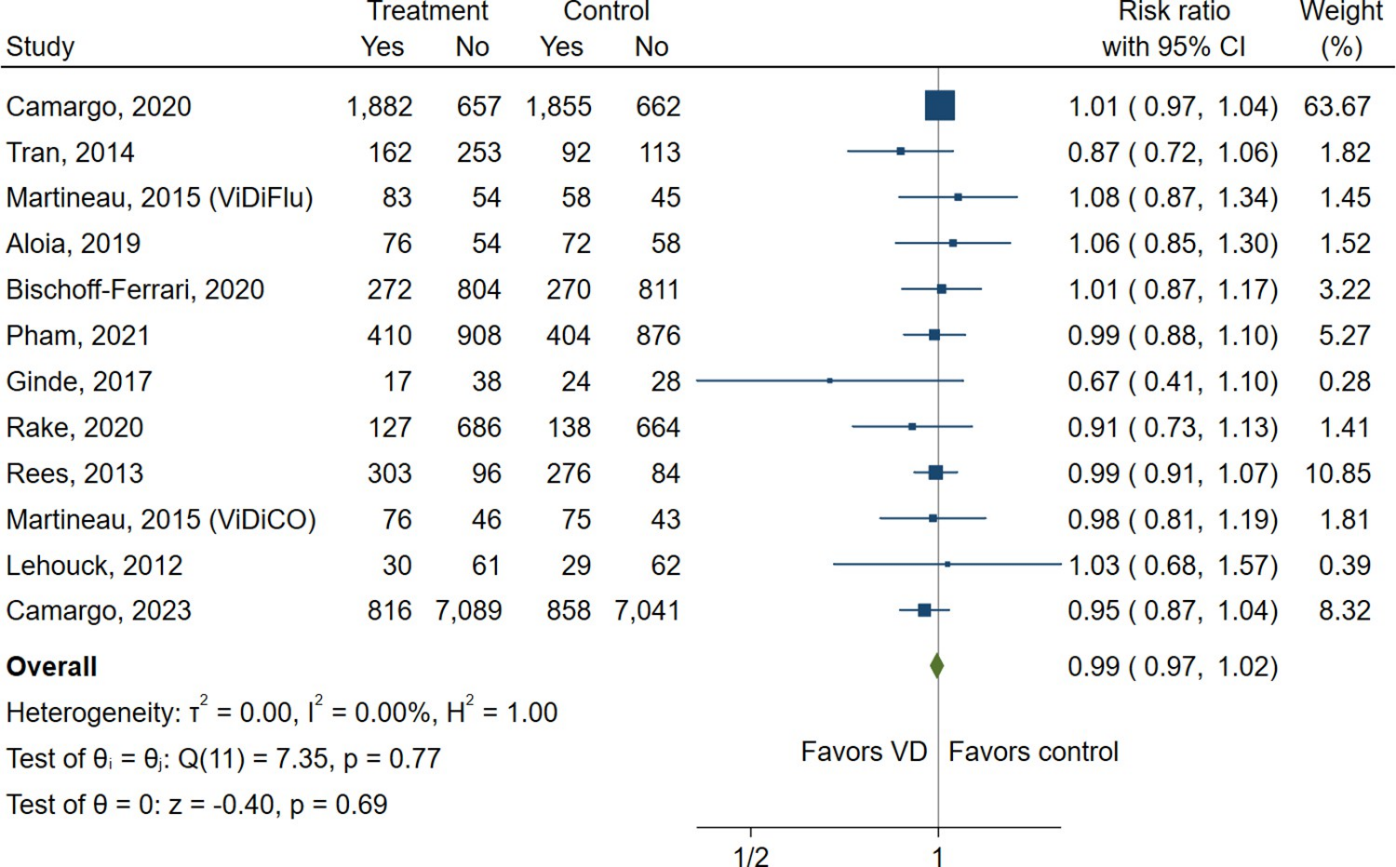
Forest plot of the meta-analysis of vitamin D supplementation in prevention of ARIs. CI, confidence interval; VD, vitamin D.

Twelve trials enrolling 29638 patients reported data for proportion of participants who had one or more ARIs. It showed that vitamin D supplementation probably does not reduce the incidence of ARIs (RR, 0.99; 95% CI, 0.97–1.02, I^2^ = 0%; moderate certainty; Fig 2) between vitamin D supplementation and control group. Eight studies including 26932 patients evaluated the incidence of URIs, and LRIs were determined in four trials enrolling 9509 patients. The results showed that vitamin D supplementation likely does not reduce URIs (RR, 1.00; 95% CI, 0.97–1.03, I^2^ = 2.0%, moderate certainty) and LRIs (RR, 0.99; 95% CI, 0.91–1.07, I^2^ = 0%, moderate certainty) (S1 Fig in S1 File). Meta-analysis of four trials indicated that vitamin D supplementation probably results in little to no difference in hospital admission due to an ARI (RR, 0.98; 95% CI, 0.87–1.09, I^2^ = 0%, moderate certainty) (S1 Fig in S1 File). The funnel plot did not show evidence of publication bias visually (S2 Fig in S1 File)

### 3.4 Subgroup and sensitivity analysis

**Fig 3:**
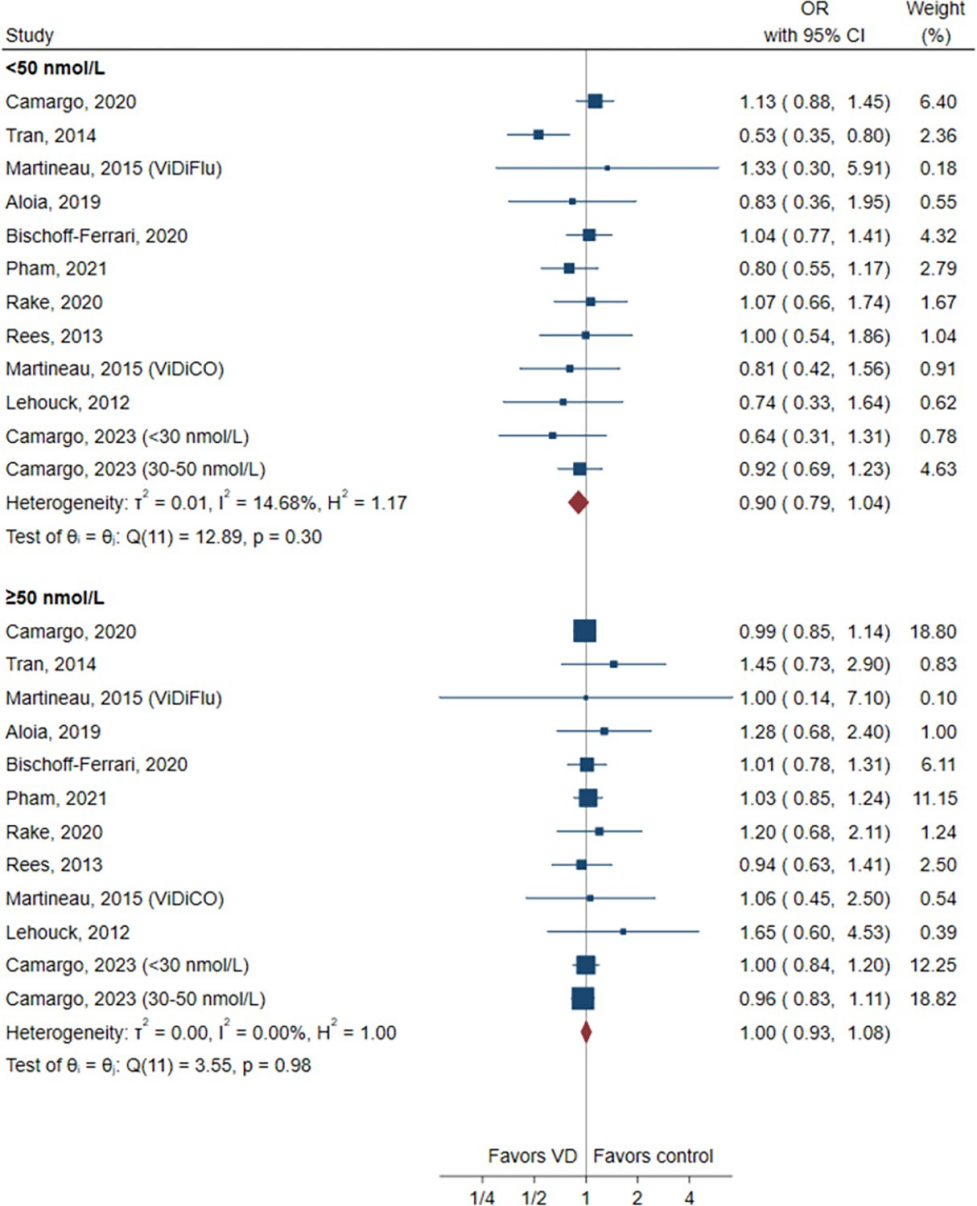
Forest plot of subgroup analysis by baseline 25(OH)D concentration. CI, confidence interval; VD, vitamin D; OR, odds ratio.

We performed subgroup analysis for the primary outcome by baseline 25(OH)D concentration (<50 nmol/L vs ≥ 50 nmol/L) (Fig 3). Although the difference was not statistically significant, there was a suggestion that vitamin D could potentially reduce the risk of ARI in patients with a baseline 25(OH)D concentration <50 nmol/L (OR, 0.90; 95% CI, 0.79–1.04, I^2^ = 14.7%). However, we did not observe this trend in patients with a baseline 25(OH)D concentration ≥50 nmol/L (OR, 1.00; 95% CI, 0.93–1.08, I^2^ = 0%). We also conducted subgroup analysis by control treatments, dose frequency, study duration, and participants’ condition. The results revealed that there was no significant association observed in those subgroups (S1 File). Sensitivity analysis was also performed by excluding each study individually and removing studies at high risk of bias, and the results were comparable to those of the main analysis.

### 3.5 Safety outcomes

**Fig 4:**
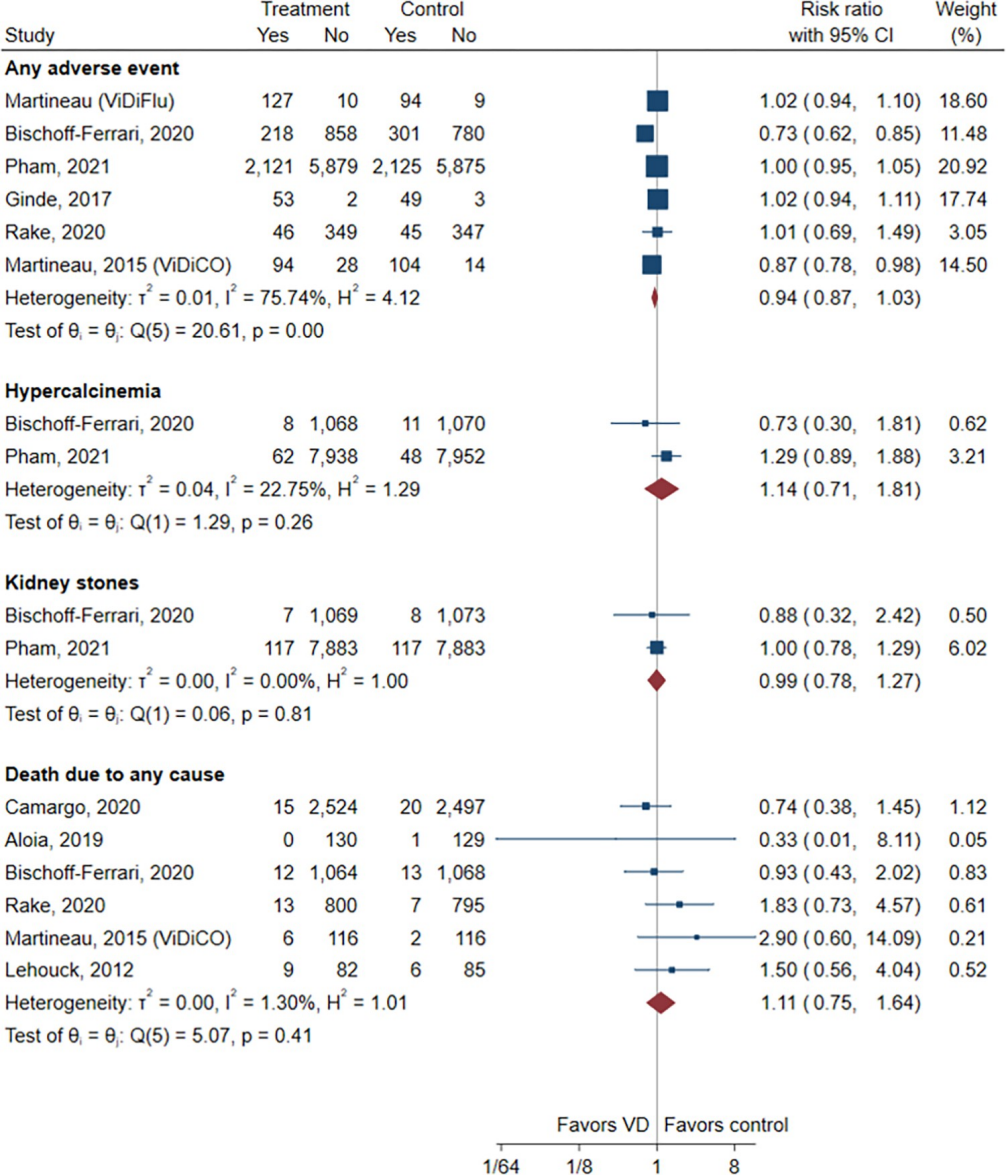
Forest plot of the meta-analysis of safety outcomes. CI, confidence interval; VD, vitamin D.

The results showed that vitamin D supplementation may result in little to no difference in death due to any cause (6 trials; RR, 1.11; 95% CI, 0.75–1.64, I^2^ = 1.3%; low certainty), any adverse event (6 trials; RR, 0.94; 95% CI, 0.87–1.03, I^2^ = 75.7%; low certainty), hypercalcemia (2 trials; RR, 1.14; 95% CI, 0.71–1.81, I^2^ = 22.8%; moderate certainty), and kidney stones (2 trials; RR, 0.99; 95% CI, 0.78–1.27, I^2^ = 0.0%; moderate certainty) (Fig 4).

## 4 Discussion

This meta-analysis included 12 RCTs involving 43118 participants and provided an overview of the efficacy and safety of vitamin D supplementation in preventing ARIs in older adults. Moderate certainty evidence indicated that there was no difference in the incidence of ARIs between vitamin D supplementation group and control group among older adults. The result was consistent in subgroup analysis by type of ARIs. For safety outcomes, the use of vitamin D supplements has no significant difference in death due to any cause, any adverse event, hypercalcinemia, and kidney stones. While two trials were classified as high risk and three trials had some concerns, it is worth noting that the sample sizes in those trials were small. Furthermore, the results of the sensitivity analysis suggest that the primary outcome remains stable and reliable.

Many observational studies have investigated the association of vitamin D concentrations and ARIs. A cross-sectional study conducted in Australia found that after adjusting for age, gender, season, smoking, BMI, physical activity, and respiratory disease history, vitamin D deficiency (<20 ng/mL) was an independent risk factor for bronchitis [34]. The NHANES study confirmed that a 25(OH)D level below 30 ng/mL was associated with a 58% increased risk of ARIs [35]. This is consistent with the findings of a meta-analysis of observational studies, which showed a positive correlation between vitamin D deficiency and disease severity [8]. Furthermore, vitamin D deficiency was identified as an independent risk factor for community-acquired pneumonia (CAP), increasing the risk by 64% [36,37]. In patients hospitalized with CAP and acute respiratory distress syndrome, vitamin D deficiency was observed in 85% to 95.4% of cases and was positively correlated with disease severity, total hospitalization days, and ICU stay, but not with in-hospital and 6-month mortality rates [38,39]. It is worth noting that a data analysis of COVID-19 in 20 European countries revealed a negative correlation between vitamin D levels and the number of cases and mortality rates [40].

Many previous meta-analyses of RCTs concluded that vitamin D supplementation could reduce the risk of ARIs [10,11,41,42]. But all of them enrolled participants irrespective of age. While Jolliffe’s review primarily indicated a reduction in the risk of ARIs with vitamin D supplementation, this protective effect was not evident among individuals aged 65 or older (OR, 0.96; 95%CI, 0.90–1.02), aligning with our findings [10]. Recognizing the limited analysis for older adults in Jolliffe’s review, our study offers a more comprehensive examination, encompassing more than double the number of participants included in Jolliffe’s review. Recently, a review by Cho and colleagues showed that vitamin D supplementation has no clinical effect in the prevention of ARIs in the main analysis and subgroup analysis of high-quality studies [12], which was consistent with our study. Some reviews also explored the association between vitamin D supplementation in children and the risk of ARIs [13,14,43,44], all of them indicated that vitamin D supplementation provided no benefit in preventing ARIs. However, two of these reviews showed high-dose vitamin D might reduce the incidence of influenza in children [13,14]. Another review also suggested that the most effective approach to optimizing and sustaining serum (25(OH)D) concentration throughout the year is by administering a significant bolus dose, followed by monthly or bi-monthly maintenance doses [45].

This is the first comprehensive meta-analysis to investigate the efficacy and safety of vitamin D supplementation in prevention of ARIs among older adults. This review has several strengths. Firstly, we conducted a comprehensive search of multiple databases and included all available RCTs of vitamin D supplementation for older adults. Secondly, the quality of evidence for each outcome was evaluated using GRADE. Thirdly, subgroup analyses were performed to explain heterogeneity, and sensitivity analysis was conducted to examine the robustness of our findings. Our conclusions are based on a large sample of approximately 43118 randomized participants, which increases generalisability. This study also has some limitations. First, the included studies varied in regimens, doses, duration, center settings, and participants’ characteristics, leading to increased clinical heterogeneity and statistical heterogeneity in some analysis. Second, due to the unavailability of data in the included primary studies, we were unable to evaluate the differences in the preventive effect on ARIs between individuals with vitamin D deficiency and those with normal vitamin D levels. It would be optimal to examine the efficacy of vitamin D supplementation in preventing ARIs considering the baseline levels of 25(OH)D, because many observational studies indicated the association of vitamin D deficiency and respiratory infections. Third, our analysis was based on study-level data rather than individual patient data, which limited the power of our analysis and the investigation of potential effect-modifiers. Fourth, the preventive effect of vitamin D supplements on ARIs was not the primary outcome in several included trials. Finally, our search was limited to studies published in English, and the inclusion of trials solely from high-income countries in this meta-analysis emphasizes the necessity for more evidence from low- and middle-income countries. This will help improve the generalizability of the findings and provide a more comprehensive understanding of the topic.

## 5 Conclusions

In conclusion, this systematic review and meta-analysis indicates that vitamin D supplementation among older adults probably results in little to no difference in the incidence of ARIs. However, further evidence is needed, particularly for individuals with vitamin D deficiency and populations residing in low and middle income countries.

## Supporting information

S1 Checklist PRISMA 2020 checklist. (DOCX)

S1 File Supplementary material. (DOC)

S1 Data (XLS)
