# Supplementary material for: Vitamin D supplementation for prevention of acute respiratory infections in older adults: A systematic review and meta-analysis
Source: PLoS One. 2024 May 24;19(5):e0303495. doi: 10.1371/journal.pone.0303495 (PMC11125479; doi:10.1371/journal.pone.0303495)
Supplement: S1 File — (DOC) [file pone.0303495.s002.doc]

## Supplementary Material

**Vitamin D Supplementation for Prevention of Acute Respiratory Infections in Older Adults: A Systematic Review and Meta-analysis**

Hao Jia, Feng Sheng, Yulan Yan, Xiaozhi Liu, and Baoqi Zeng

**Content**

**Search strategy**

S1 Fig. Forest plot of the meta-analysis of vitamin D supplementation in prevention of upper respiratory infection, lower respiratory infection, and hospital admission due to an acute respiratory infection.

S2 Fig. Funnel plot for primary outcome.

S3 Fig. Subgroup analysis by control treatments (placebo versus lower-dose) for primary outcome.

S4 Fig. Subgroup analysis by dose frequency (daily vs bolus vs combination) for primary outcome.

S5 Fig. Subgroup analysis by study duration (≤ 1 year vs > 1 year) for primary outcome.

S6 Fig. Subgroup analysis by participants’ condition (healthy vs comorbidity) for primary outcome.

### Search strategy

**Search strategy for Embase (814 items)**

1. 'vitamin d':ti,ab OR 'vitamin d2':ti,ab OR 'vitamin d3':ti,ab OR '25-hydroxyvitamin d':ti,ab OR 'vitamin d supplements':ti,ab OR 'vitamin d supplementation':ti,ab OR '1alpha, 25-dihydroxyvitamin d':ti,ab
2. holecalciferol:ti,ab OR ergocalciferol:ti,ab OR alphacalcidol:ti,ab OR alfacalcidol:ti,ab OR calcitriol:ti,ab OR paricalcitol:ti,ab OR doxerocalciferol:ti,ab
3. 'vitamin d'/exp
4. #1 OR #2 OR #3
5. 'respiratory tract infection*':ti,ab OR 'acute respiratory infection*':ti,ab OR copd:ti,ab OR pneumonia:ti,ab
6. 'covid-19':ti,ab OR 'sars-cov-2':ti,ab
7. infectious:ti OR infection*:ti
8. #5 OR #6 OR #7
9. trial*:ti,ab OR random*:ti,ab OR placebo*:ti,ab OR 'controlled clinical trial'/exp
10. #4 AND #8 AND #9

**Search strategy for PubMed (522 items)**

1. Vitamin D [MESH]
2. ('vitamin d' OR 'vitamin d2' OR 'vitamin d3' OR '25-hydroxyvitamin d' OR 'vitamin d supplements' OR 'vitamin d supplementation' OR '1alpha, 25-dihydroxyvitamin d') [Title/Abstract]
3. (holecalciferol OR ergocalciferol OR alphacalcidol OR alfacalcidol OR calcitriol OR paricalcitol OR doxerocalciferol) [Title/Abstract]
4. #1 OR #2 OR #3
5. ('respiratory tract infection*' OR 'acute respiratory infection*' OR copd OR pneumonia) [Title/Abstract]
6. ('covid-19' OR 'sars-cov-2') [Title/Abstract]
7. (infectious OR infection*) [Title]
8. #5 OR #6 OR #7
9. Controlled Clinical Trial [MESH]
10. (trial* OR random* OR placebo*) [Title/Abstract]
11. #9 OR #10
12. #4 AND #8 AND #11

**Search strategy for Cochrane Library (484 items)**

1. [mh "Vitamin D"]
2. ("vitamin d" or "vitamin d2" or "vitamin d3" or "25-hydroxyvitamin d" or "vitamin d supplements" or "vitamin d supplementation" or "1alpha, 25-dihydroxyvitamin d"):TI,AB
3. (holecalciferol or ergocalciferol or alphacalcidol or alfacalcidol or calcitriol or paricalcitol or doxerocalciferol):ti,ab
4. #1 OR #2 OR #3
5. ("respiratory tract infection*" or "acute respiratory infection*" or copd or pneumonia):ti,ab
6. ('covid-19' or "sars-cov-2"):ti,ab
7. (infectious or infection*):ti
8. #5 OR #6 OR #7
9. #4 AND #8

**Search strategy for Clinicaltrial.gov (61 items)**

"vitamin d" OR "vitamin d2" OR "vitamin d3" OR "25-hydroxyvitamin d" OR "vitamin d supplements" OR "vitamin d supplementation" OR "1alpha, 25-dihydroxyvitamin d" | infection* OR covid-19 OR sars-cov-2

### Supplementary Figures


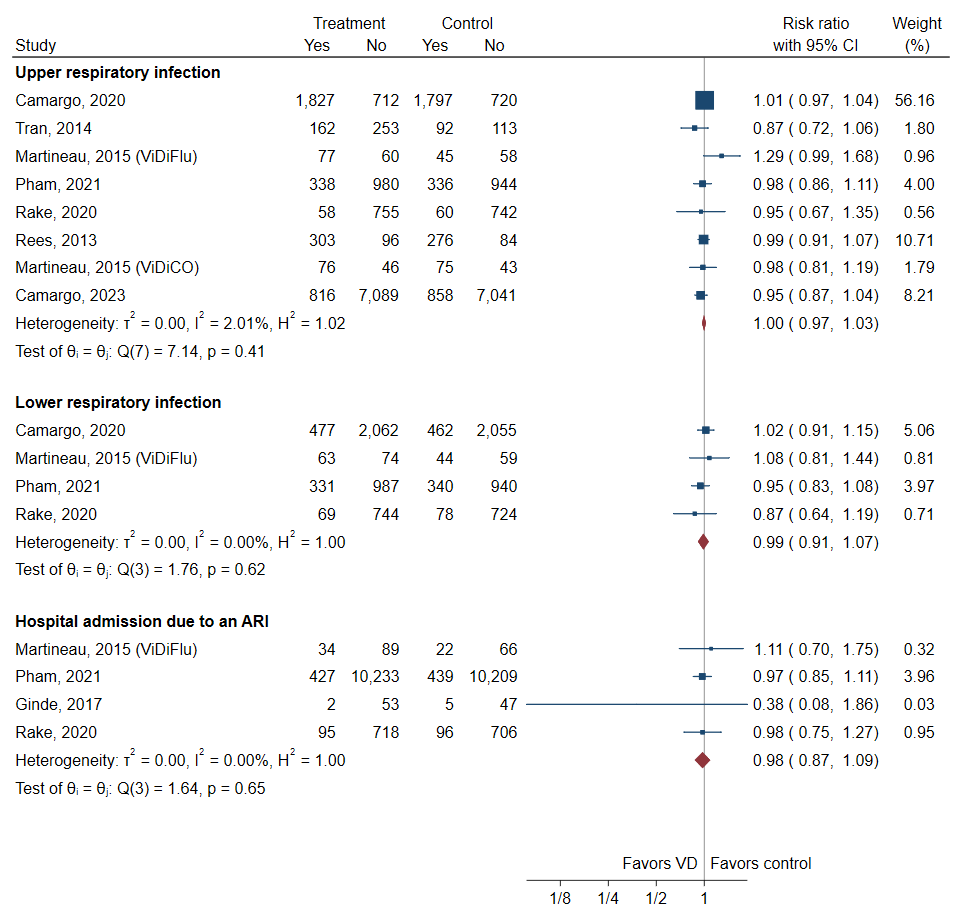


**S1 Fig. Forest plot of the meta-analysis of vitamin D supplementation in prevention of upper respiratory infection, lower respiratory infection, and hospital admission due to an acute respiratory infection.**


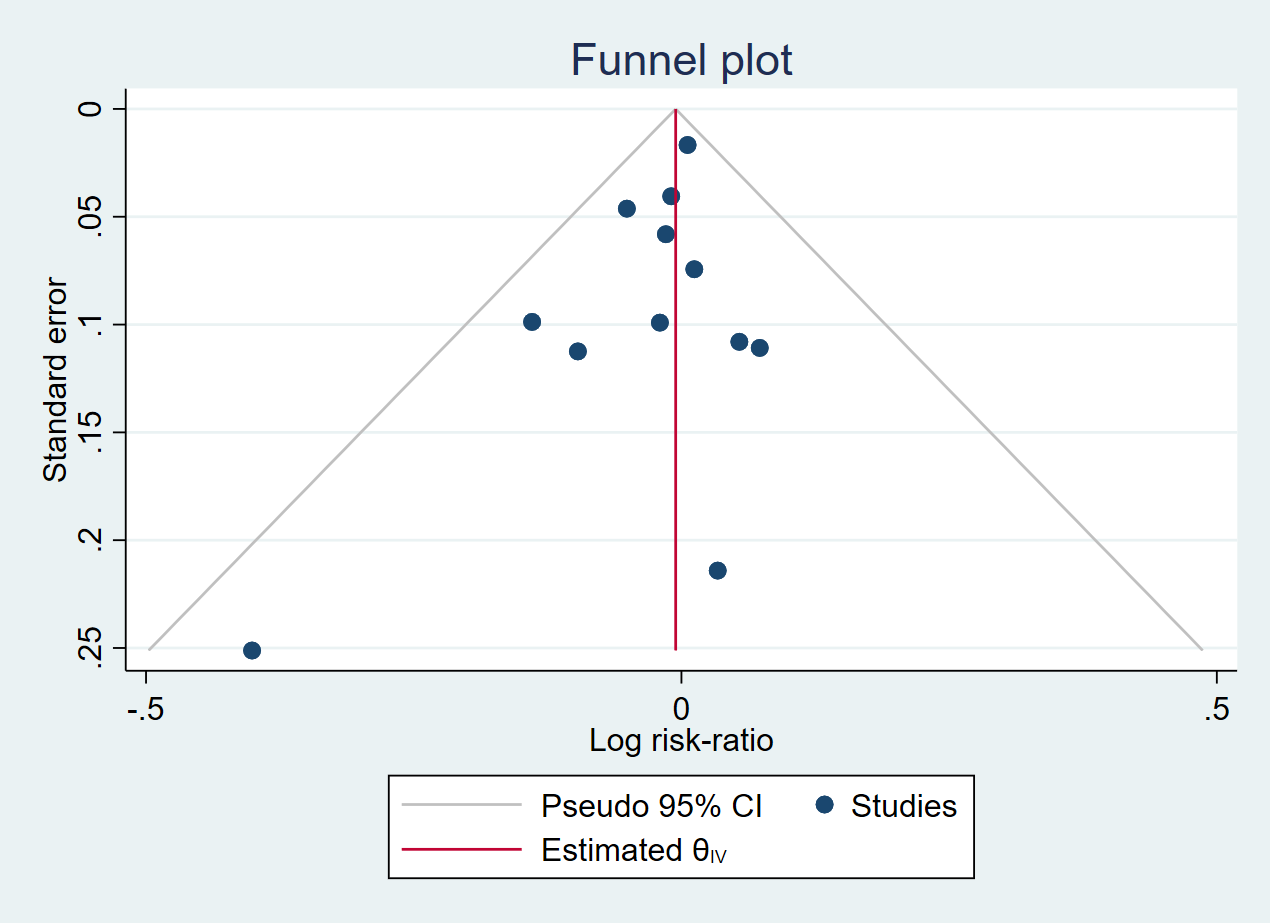


S2 Fig. Fu**nnel plot for primary outcome.**


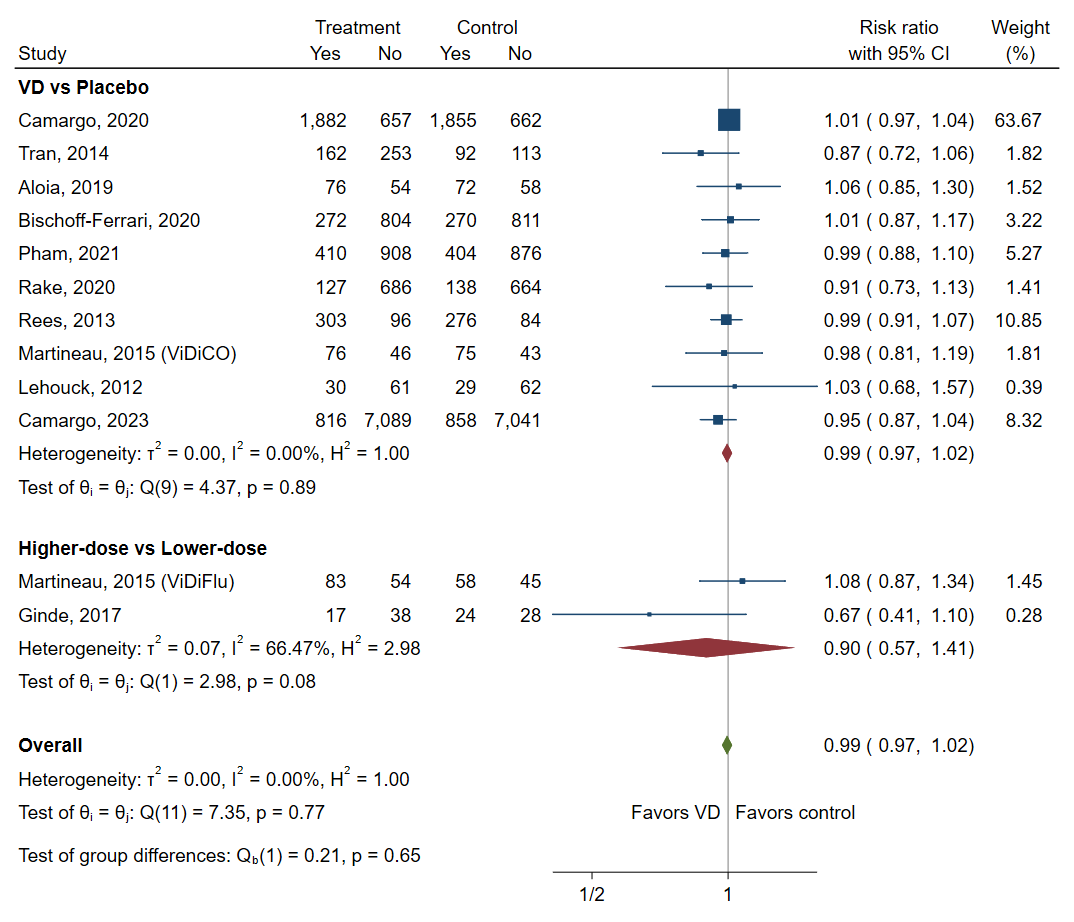


S3 Fig. **Subgroup analysis by control treatments (placebo versus lower-dose) for primary outcome.**


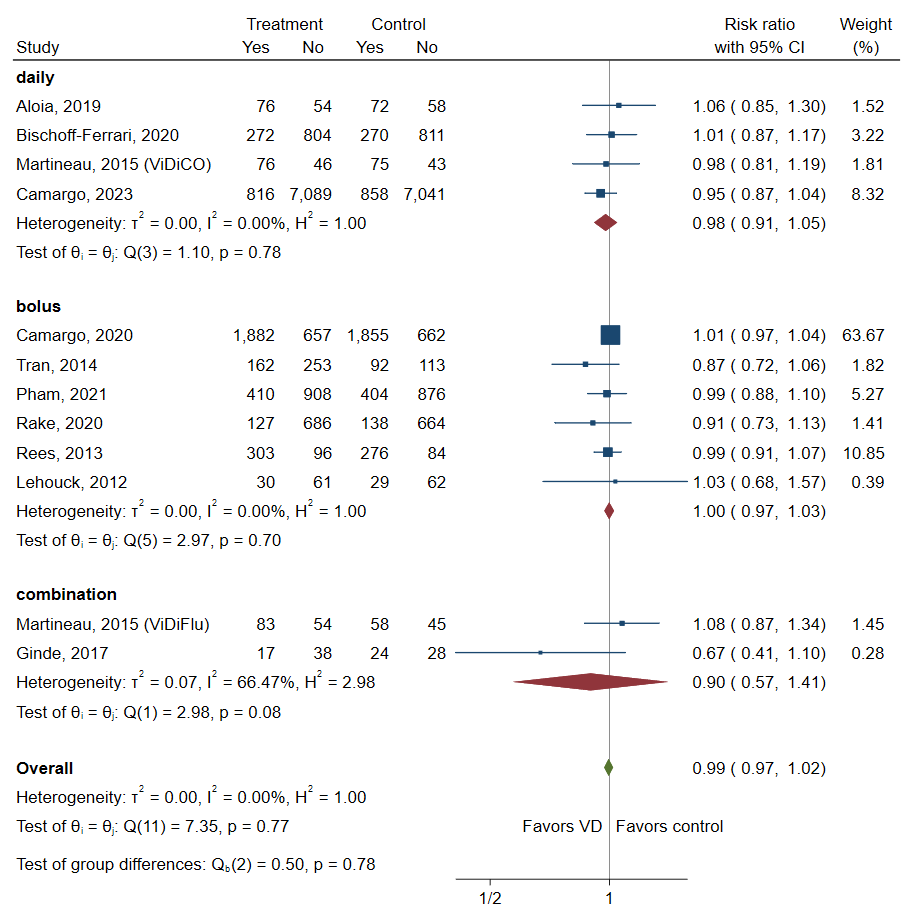


S4 Fig. **Subgroup analysis by dose frequency (daily vs bolus vs combination) for primary outcome.**


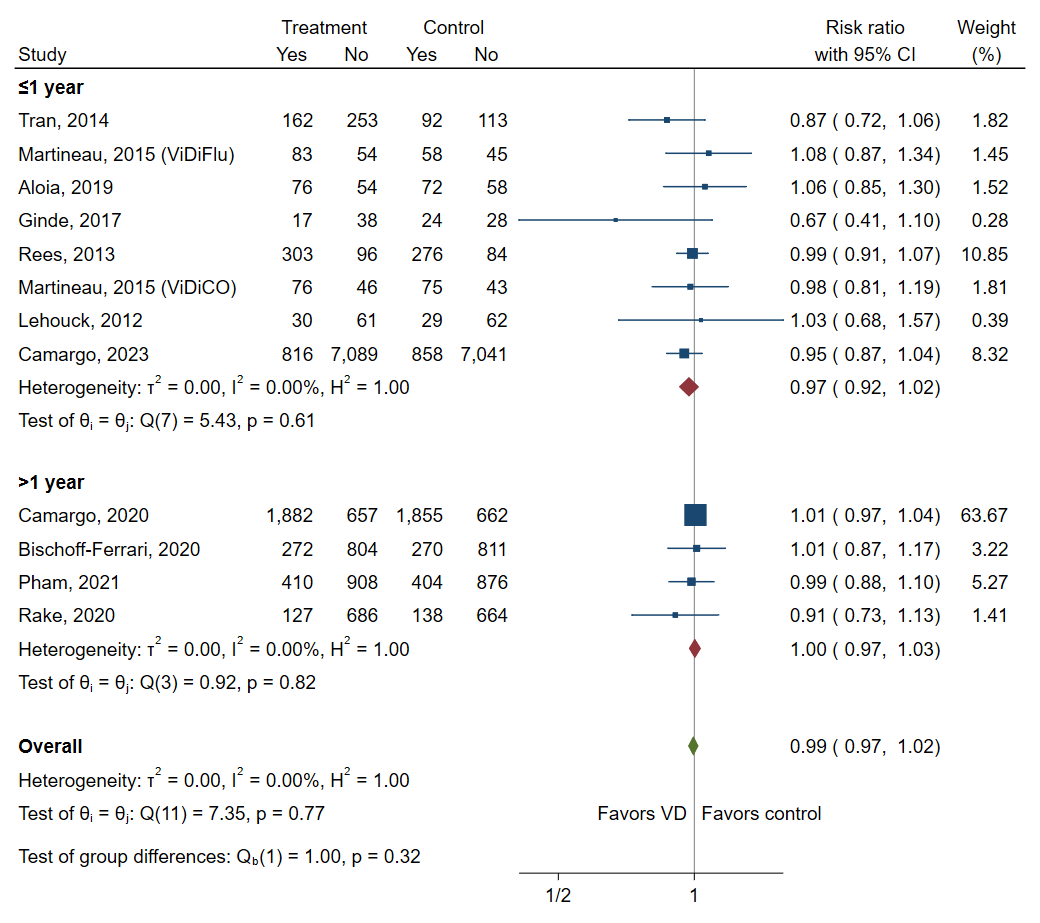


S5 Fig. **Subgroup analysis by study duration (≤ 1 year vs > 1 year) for primary outcome.**


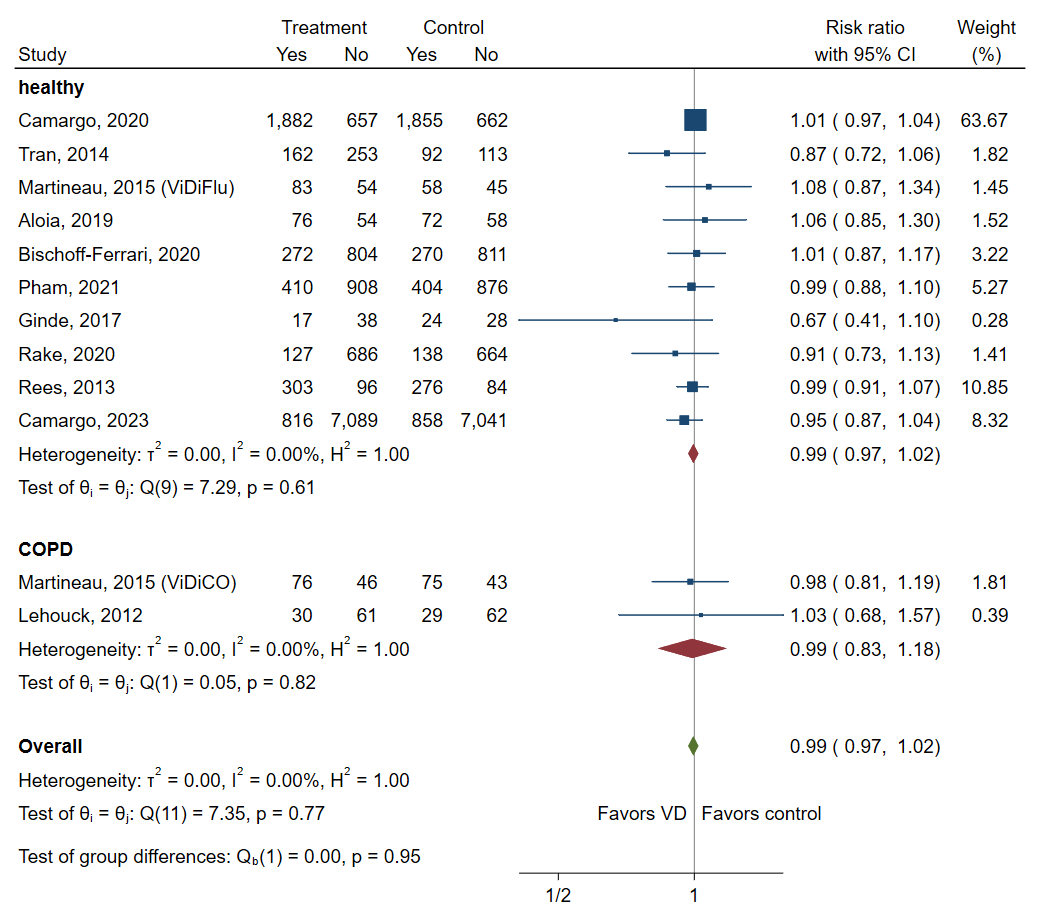


S6 Fig. **Subgroup analysis by participants’ condition (healthy vs comorbidity) for primary outcome.**
